# Supplementary material for: Are carbon emissions trading and green financial instruments synergistic? -Comprehensive quantitative research based on content analysis
Source: PLoS One. 2024 Mar 7;19(3):e0298601. doi: 10.1371/journal.pone.0298601 (PMC10919601; doi:10.1371/journal.pone.0298601)
Supplement: S1 Data — (ZIP) [file pone.0298601.s001.zip › Data/地级市Z_F工作报告.zip!/╣π╢1⁄2/╘╞╕í╩╨/└┤╫╘╣1⁄2╓┌║┼í╛┬φ┐╦╩2╛▌═°í┐_2019.docx]

各位代表：

我代表市政府向大会作政府工作报告，请予审议，并请政协委员和其他列席人员提出意见。

2018年工作回顾

2018年，是全面贯彻党的十九大精神的开局之年，是改革开放40周年，是决胜全面建成小康社会的关键一年。在习近平新时代中国特色社会主义思想指导下，在省委省政府和市委的正确领导下，在市人大及其常委会和市政协的监督支持下，市政府全面落实习近平总书记对广东工作重要指示批示和省委十二届历次全会部署，以及市第六次党代会和市委六届三次、四次全会精神，围绕打造“两新一前列”、促进实现“美丽云浮、共同缔造”的战略定位，

坚持新发展理念，贯彻高质量发展要求，经济社会发展取得新成绩，较好地完成了市六届人大三次会议确定的目标任务。

2018年，全市经济发展稳中有进。预计全市实现地区生产总值851.1亿元，同比增长（下同）3.6%；固定资产投资总额增长11%；地方一般公共预算收入57.64亿元，增长0.3%；税收总收入110.58亿元，增长8.4%；社会消费品零售总额385.75亿元，增长8%;实际利用外资3863.9万美元，增长37.5%；城乡居民人均可支配收入增长6.3%；12月末，全市金融机构本外币存款余额1199.5亿元、贷款余额793.07亿元，分别增长7.02%、8.42%。经济结构继续优化，三次产业比重调整为17.8∶37.9∶44.3。

一、现代产业体系建设有新进展

传统产业加快转型升级。全市新增工业企业技术改造备案项目64个，总投资25.06亿元。水泥、石材等产业提质增效，税收分别增长105.1%、45%。第十五届云浮国际石材科技展览会暨第九届云浮石文化节参观人数、成交额分别增长48.3%、5.3%。全国罗定绿色稻米产业知名品牌创建示范区、全国不锈钢餐厨具产业知名品牌创建示范区、国家地理标志产品保护示范区(广东罗定)创建工作基本完成，全国云浮石材产业知名品牌创建示范区创建工作进展顺利。新增广东省名牌产品（工业类）3个。被评为“全省促进中小微企业上规模工作示范市”。组建云浮市企业上市促进会和新三板投资平台。益坤农业、新云石业等上市后备企业启动股改，天露山旅游度假区有限公司、稻香园农业发展有限公司等企业完成股改。

新兴产业加快培育。氢能小镇累计引进全产业链项目21个，总投资51亿元。佛山（云浮）产业转移工业园获批筹建国家级氢能产业技术标准创新基地。佛山（云浮）氢能产业与新材料发展研究院获批广东省氢能技术重点实验室。国鸿氢能获批广东省工程技术研发中心。国鸿氢能燃料电池占工信部公布的燃料电池汽车目录份额的46%。氢能物流车样车完成试制，氢燃料电池移动应急电源车研发成功。新增加氢站1座，在建9座。云计算数据中心一期基本建成，华为公司首批约500个机柜已部署到位。“核高基①”重大专项取得突破，我市成为全省唯一党政机关电子公文系统安全可靠应用非涉密领域试点。“广东（云浮）安全可靠系统应用与产业示范区”加快构建，龙芯中科、安可云、龙启智能、正盟科技落户云浮。市健康医药产业园一期建设进展顺利，香港衍生集团、广东颂春药业、广东康恩中医药等项目加快建设，二期完成征地2200亩并动工建设。广东禅文化创意产业园等一批旅游项目加快建设，新兴县获评广东2017-2018年度旅游综合竞争力十强县。

发展载体加快完善。全市共引进合同项目120个，计划总投资412.5亿元。引进万洋众创城、邦诚科技等投资超10亿元合同项目12个。罗定产业转移集聚地、郁南产业转移集聚地被确认为省产业转移工业园，云城产业集聚地扩园获省批准。国际现代农牧装备产业园动工建设。云浮新区云联小镇、新兴温氏农科小镇纳入省级特色小镇培育库。

重点项目建设扎实推进。53项省重点项目完成投资114.44亿元，占年度计划投资的95.95%；142项市重点项目完成投资202.72亿元，占年度计划投资的88.11%。华润西江发电厂项目前期工作取得重大突破，罗定华耀科技等24个项目建成投产。

创新能力不断增强。全市新增高新技术企业19家，总数达到58家。新增省级新型研发机构2家，总数达到4家。新建云浮中科石材创新中心等创新平台。创建广东国际人才交流与创新中心云浮区域中心。温氏集团两项科技成果获2018年度国家科学技术进步奖二等奖。新增国家级博士后科研工作站2家、省级博士工作站5家。

二、乡村建设打开新局面

乡村产业加快发展。广东省现代特色南药试验区加快建设，全市南药种植面积105万亩，建成南药种子种苗繁育示范基地5个，南药标准化种植示范基地60个。新增全国“一村一品”示范村1个、国家地理标志保护产品1个、广东省名牌产品（农业类）2个。云浮市（云城）南药产业园、云浮市南药（罗定肉桂）产业园、温氏新兴优质鸡产业园获批省级现代农业产业园。罗定市榃滨镇、新兴县簕竹镇获农业农村部、财政部批准开展农业产业强镇示范建设。全域旅游加快发展，打造乡村旅游点43个、乡村旅游精品线路16条，涌现了罗定长岗坡、南江传统文化特色小镇等一批有影响力的乡村旅游景点。

农村人居环境不断优化。改造拓宽农村公路478公里。90条行政村实现通快递物流，66条行政村实现通客运班车。建成169个农村电商服务站，云安区、郁南县获评省级电子商务进农村综合示范县（区）。县域乡村建设规划实现全覆盖，村庄规划编制工作有序推进。建成镇级污水处理厂5座，一批农村污水处理设施建成投入运营。农村生活垃圾清运率达98.35%，有效处理率达96.05%。全面推进农村建设用地拆旧复垦，连续18年实现耕地占补平衡。

脱贫攻坚纵深推进。累计帮扶到村项目3120个,带动贫困户1.2万户、6万多人，实现户均增收6205元。全年实现预脱贫2.3万人。全市105条省定贫困村基本完成村道拓宽和硬底化、“三清三拆三整治②”工作。省定贫困村新一轮农网改造升级全面完成。

三、城市品质有新提升

中心城区不断提质。全国文明城市创建工作深入推进。市中心城区近期建设规划范围内控规实现全覆盖。开展创建“干净、整洁、平安、有序”城区活动。北一路建成通车，金山路碧桂园段实现双边贯通，浩林西路、东方路贯通工程等44个提升中心城区首位度项目扎实推进，初步解决了一批多年想解决而未能解决的问题。完成“三旧”改造面积649.84亩。田心片区综合提升改造基本完成。英东体育场地下人防工程主体结构封顶。城镇生活垃圾无害化处理率达100%。云浮新区建设不断加快。中央商务区综合交通枢纽站前广场基本建成，市档案综合大楼主体建设完成80%，市图书馆、光明中小学、新区水厂一期、西江新城文化中心等项目加快推进。云浮新区“云谷”示范区海绵城市③试点建设完成。新城快线二期、东部快线加快建设，文华路东段建成通车。

交通基础设施日臻完善。交通基础设施完成投资51.3亿元，完成年度计划的101%。汕湛高速云湛段（云浮段）建成通车，全市高速公路通车总里程达330公里。汕湛高速清云段、高恩高速、怀阳高速二期、罗信高速等项目加快建设。完成国省道路面改造77公里。

四、生态建设取得新成效

  污染防治攻坚战深入推进。坚决按中央环保督察“回头看”、

省环保督察反馈意见落实整改。坚决打赢蓝天保卫战，全年空气质量优良率达92.3%。着力打好碧水攻坚战。南山河流域云浮城区（云城、高峰街道）截排系统完善及污水处理厂扩建项目截排系统部分主体工程基本完成，南山河永丰桥断面水质达到Ⅲ类。全市5个地表水考核断面达到省考核目标要求，综合指数排全省第3名。完成7段城市建成区黑臭水体整治。基本完成地级市以上饮用水源保护区环境问题整治。全面完成13宗山区五市中小河流治理任务。全面实施河长制、湖长制，设置河长1085人、湖长7人。扎实推进净土防御战。固体废物专项整治成效明显，建成花岗岩石材废渣综合利用项目3个，2个大理石石材废渣综合利用项目加快建设。

新生态加快建设。完成造林更新17.47万亩，完善提升生态景观林带19.5公里，完成44.34万亩森林抚育任务，完成年度森林公园建设任务，森林覆盖率达67.05%④。国有林场改革通过省验收，国家森林城市创建工作加快推进。

五、各项改革取得新突破

重点领域改革全面深化。供给侧结构性改革深入推进，全年累计依法减税52.02亿元,减免政府性基金及行政事业性收费3.22亿元。开展新一轮基础设施补短板储备工作，完成投资145.79亿元。农村综合改革加快推进，“创新农村土地股份合作经营机制”试点获农业农村部批复，郁南县农村集体产权制度改革试点上升为国家级试点。基本完成农村土地承包经营权确权登记颁证工作。金融体制改革有序推进。全市首家法人非银行金融机构温氏集团财务有限公司挂牌开业。新兴、罗定农商行完成改制。在全省率先推出无核黄皮气象灾害指数保险、农地抵押贷款、税贷通、惠农贷、“互联网+信用三农”特色农业众筹融资等一批金融创新试点。“五平台五基金⑤”累计为中小微企业提供新增贷款43.64亿元，降低融资成本3217.34万元。医药卫生体制改革全面推进，开展公立医院薪酬制度改革、医联体建设试点，取消公立医院医用耗材加成。

行政体制改革扎实推进。“放管服”改革十大行动计划实现突破，有关经验做法被收录《中国机构改革与管理》和《广东省行政体制改革与管理这五年》。行政许可和公共服务事项累计整体承诺办理时限比法定压缩70%以上。投资项目全面实行并联审批，社会投资项目、政府投资项目从立项到开工审批时限分别缩短至35个、40个工作日以内；开办企业时间缩减至5个工作日以内。成为全省首个实现工程领域“七图联审⑥”的地市。“数字政府”建设加快，“粤省事·看云浮”移动民生服务平台正式上线，205项政务服务事项实现指尖办理。成为全省首个网上中介服务超市⑦系统交付使用的地市，539项中介服务事项进驻。《基层政务公开标准体系与关键标准研制》获国家标准委立项。新兴县顺利通过省基层政务公开标准化规范化试点验收评估。“多证合一”、“企业名称自主申报”、“证照分离⑧”等商事制度改革深入推进，全年新登记市场主体23920户，增长24.42%。市县机构改革工作稳妥推进。

六、民生保障有新成绩

全市民生类支出完成160.68亿元，占一般公共预算支出74.1%。

教育事业取得新突破。仅用两年时间建成广东药科大学云浮校区一期，创造了“云浮速度”，2018年10月正式开学，填补了我市本科高等教育领域空白。教育“创现”累计投入13.02亿元，教育强镇复评完成100%，云城区、罗定市、新兴县、郁南县创建成为广东省推进教育现代化先进县（市、区）。新兴县创建成为国家级农村职业教育和成人教育示范县。广州华立科技职业学院云浮校区获准专科招生。市高级技工学校获批为“世界技能大赛建筑石雕项目中国集训基地”和“国家级高技能人才培训基地”。

卫生强市建设加快推进。市区域医疗中心加快建设，启动建设首批5个重点专科。基层医疗卫生特殊紧缺人才“千人计划”引才模式得到省充分肯定。全国基层（社区和农村）中医药工作先进单位通过复审。严重精神障碍患者救治救助5项重点指标在全省排名靠前。

文化体育服务水平逐步提升。全面实施文化强市战略。我市“农村春晚”入选全国文化信息资源共享工程“百姓大舞台”品牌项目。县级融媒体中心加快建设，广播电视村村通向户户通升级不断推进。“扫黄打非”进基层实现全覆盖。体育场地设施基本实现全覆盖，1.2万个座席的市体育场项目基本完成。成功举办第五届“市长杯”足球联赛。我市朱明叶在世界杯击剑赛中勇夺女子重剑个人冠军；曾思妮勇夺残疾人公路自行车世锦赛一金一银。

社会保障更加完善。全市城镇新增就业2.06万人，扶持创业1626人，城镇登记失业率为2.47%。城乡低保、特困人员供养、医疗救助等补助标准稳步提高。城乡居民基本养老保险和基本医疗保险参保率均超99%。城乡居民基础养老金提高至每人每月148元，城乡居民医保最高报销限额提高至40万元。省保障农民工工资支付考核我市被评为A级优秀等次。

社会治理不断深化。深化平安云浮、法治云浮、和谐云浮建设，强力推进扫黑除恶专项斗争、“飓风2018”专项行动和全民禁毒工程，严厉打击涉黑恶、涉黄赌毒等各类突出违法犯罪。我市成为全省六个打造广东新时代“枫桥经验⑨”实践创新示范点之一，成为全省唯一成功申报2019年国家“雪亮工程⑩”公共安全视频监控建设联网应用重点支持城市。市、县两级青少年法治教育实践基地全面建成，在全省率先完成人民陪审员选任工作。深化信访制度改革，一批信访突出问题得到妥善解决。加强退役军人服务管理工作。有效应对“艾云尼”、“山竹”等重大自然灾害，非洲猪瘟等生产安全防范成效明显。认真贯彻军民融合发展战略，国防动员和后备力量建设得到加强。

七、政府建设有新气象

法治建设进一步加强。自觉接受人大法律监督和政协民主监督，认真听取民主党派、工商联、无党派人士及工会、共青团、妇联等人民团体意见建议，办理省、市人大代表建议103件，省、市政协提案190件，办结率100%。全面加强依法行政。《云浮市畜禽养殖污染防治条例》获省人大常委会表决通过，将于今年3月1日起正式实施。《云浮市石材生产加工环境污染防治条例（草案）》已提交市人大常委会初审。《云浮市城市公园和广场管理办法》正式实施。

政府作风建设取得明显成效。廉洁政府建设深入推进，全面彻底肃清李嘉、万庆良恶劣影响，“四风”整治不断深化，廉政建设责任制严格落实。审计监督、财政评审不断强化，财政资金使用和政府性投资效益持续提升。有力有序化解政府债务风险，全市债务风险总体安全可控。“三公”经费和会议费支出连续六年实现“零增长”，政风持续好转。

与此同时，民宗、外事侨务、港澳台、人防、统计、地方志、粮食、消防、打假、档案、保密、科协、侨联、文联、残联、社科联、贸促、慈善、老区、老龄以及驻市外机构等工作取得新成绩。

各位代表！过去一年的成绩来之不易。这是省委、省政府和市委正确领导、科学决策的结果，是全市人民齐心协力、奋力拼搏的结果。在此，我代表市人民政府，向全市人民，向人大代表、政协委员，向所有关心支持云浮的同志们、朋友们，致以崇高的敬意和衷心的感谢!

在总结成绩的同时，我们也充分认识到当前发展存在的问题和不足：发展速度较慢，经济总量在全省靠后的状况未得到改变；传统产业发展质量不够高，战略性新兴产业发展未形成规模效应；乡村振兴短板仍较突出，脱贫攻坚任务重；生态环保历史欠账多，污染防治形势依然严峻；中心城区首位度不高，教育、医疗等优质资源不足；营商环境还有待进一步优化；政府自身建设还存在不足。对这些问题，我们一定高度重视，认真加以解决。

2019年工作安排

2019年是中华人民共和国成立70周年，2020年是全面建成小康社会之年，2021年是中国共产党成立100周年。进入重要的历史时刻，市委六届四次、五次全会发出了要以新担当新作为开创云浮工作新局面的号召。我们要提高站位，抓准工作立足点与发力点：

——更加注重政治引领。必须坚持以习近平新时代中国特色社会主义思想为指导，深入学习贯彻习近平总书记重要讲话精神，牢固树立“四个意识”，增强“四个自信”，坚定践行“两个维护”。坚持党的领导，确保中央大政方针、省委省政府决策部署和市委工作要求落地生根。

——更加注重高质量发展。坚持传统产业转型升级和新兴产业培育“双轮驱动”，集中力量加厚底板、补强短板，提升产业层次和水平，推动经济发展质量变革、效率变革、动力变革，努力在质的显著提升中实现量的有效增长。

——更加注重改革创新。牢牢把握改革开放关键一招，持续优化行政审批制度改革，进一步深化对外开放，全方位优化营商环境。坚持创新是第一动力，统筹推进以科技创新为核心的全面创新，努力推动经济增长由投资驱动向创新驱动转变、从外延式扩张向内涵式增长转变。

——更加注重生态优先。坚持“生态立市”、“绿色崛起”发展理念，充分发掘和利用好云浮的生态优势，在高水平保护中实现高质量发展。

——更加注重为民利民。牢固树立“以人民为中心”发展理念，大力实施乡村振兴战略，坚决打赢脱贫攻坚战，努力补齐民生发展短板，改善人民群众生活质量，促进城乡协调发展。

今年市政府工作总体要求是：坚持以习近平新时代中国特色社会主义思想为指导，深入学习贯彻总书记对广东重要讲话精神，按照省委省政府和市委的部署要求，坚持稳中求进工作总基调，全面贯彻新发展理念，聚焦高质量发展，聚力抓产业兴乡村，统筹做好稳增长、促改革、调结构、惠民生、防风险各项工作，进一步稳就业、稳金融、稳外贸、稳外资、稳投资、稳预期，坚决打好防范化解重大风险、精准脱贫、污染防治“三大攻坚战”，立足“一区”、融入“一核”、协同“一带”，打造环珠三角经济带发展新引擎、粤北生态建设发展新高地，推动乡村振兴走在全省前列，促进实现“美丽云浮、共同缔造”。

2019年全市经济社会发展主要预期目标是：地区生产总值增长5.5%左右，在实际执行时努力争取更好结果；规模以上工业增加值增长5%左右；固定资产投资增长8.5%；地方一般公共预算收入增长6%以上；社会消费品零售总额增长6.5%左右，居民人均可支配收入增长和经济增长基本同步。推进135项重点项目建设，计划总投资1114.51亿元，年度计划投资170.23亿元。围绕上述目标，重点抓好七方面工作。

一、大力发展实体经济，打牢高质量发展的根基

推动制造业高质量发展。牢牢扭住制造业高质量发展这个核心，带动整个工业体系高质量发展。认真落实省“实体经济新十条”和市“石材十条”、“不锈钢制品十条”、“硫化工十条”等政策措施，加快推动传统制造业优化升级。推进全国云浮石材产业知名品牌创建示范区建设。大力发展石材机械制造业，培育一批石材机械龙头企业。大力发展现代农牧装备制造业，加快推动国际现代农牧装备产业园建设。加快培育新兴产业，推动新兴装备制造业创新发展。大力发展氢能源装备制造业，加快建设广东省氢能技术重点实验室，加快加氢站建设和氢能源汽车市场推广，推动氢能源产业市场化应用实现新突破。大力发展云计算大数据产业，继续抓好“核高基”重大专项示范应用，加快推动省市共建安全可靠产业基地建设。

全力支持民营经济发展。认真贯彻落实省“民营经济十条”等惠企政策，实施重点民营企业帮扶行动计划，帮助民营企业减负去荷、减压纾困。发挥好“五平台五基金”的作用，解决民营企业融资难融资贵问题，力争扶持1～2家民营企业股改上市。加快发展现代金融业，推动现代物流、科技服务、信息技术服务、商务服务等生产性服务业发展。

大力引进优质项目。按照“财税贡献大、科技含量高、生态环保、集约节约用地、产业带动力强”原则，积极引进一批优质项目，确保实现全年招商引资400亿元的目标任务。加快推进华润西江发电厂、金盛兰集团年产800万吨优特钢、碧桂园新型材料、嘉士利黄皮小镇、万洋众创城等项目建设。加快全市5个省级产业转移园（产业集聚地）和云计算大数据、健康医药、绿色日化等专业园区建设，加快开发腰古片区，推动优质项目加速集聚。

加强创新能力建设。加快培育高新技术企业，年内新增高新技术企业8家以上，省高新技术企业培育入库企业8家以上。加快新型研发机构、重点实验室建设，规模以上工业企业建立研发机构覆盖率达22%。培育一批科技企业孵化器、工程技术研究中心，加快建设云浮国际创新院，积极推进广东国际人才交流与创新中心云浮区域中心建设，加快创建国家高新区。大力引进培养优秀人才，继续实施引进高层次及基层医疗卫生特殊紧缺人才“千人计划”。

二、全力推进乡村振兴，激活高质量发展的潜力板

加快省级现代农业产业园建设。争取年内新增省级现代农业产业园2～3个。大力发展道地南药产业，加快建设广东省现代特色南药试验区。抓好育种育苗基地、生产加工基地和物流仓储平台、技术成果转化平台“两基地、两平台”建设。力争年内创建7个“一镇一业”示范镇、35条“一村一品”示范村。积极开展国家地理标志产品申报保护工作。升级温氏“现代农业+”和凌丰“现代工业+”模式，推动农村一二三产业融合发展。开展新一轮农网改造升级，加快推进220千伏云城硫都、罗定黎少等重点输变电工程和新兴农村智能电网示范区建设。

加快推进行政村村道改造。大力推进全市“四好”农村路建设，完成550公里农村公路路面硬底化和300公里行政村村道拓宽改造任务，加快推进自然村村道改造。

加快推进乡村通物流通电商。鼓励发展乡村物流业，打通镇村到农户“最后一公里”物流快递瓶颈。积极发展农村电商，加快培育一批电商龙头企业，谋划建设云浮市电子商务服务中心。

加快推进“三清三拆三整治”。实施“十村精品、百村示范、千村整治”乡村人居环境提升计划，所有纳入规划的村庄全部完成农村人居环境综合整治任务。加快推进乡村规划编制工作，年内基本完成村庄规划编制。

提高农村生活垃圾和污水处理水平。加快推进农村生活污水收集处理设施建设，大力推进农村改水改厕工作。推动农村生活垃圾无害化、减量化和资源化利用，提高生活垃圾处理水平。推动省定贫困村全部建成省卫生村或市卫生村。

积极谋划和推动全域旅游。编制《云浮市全域旅游发展规划》和《云浮市西江流域绿色生态旅游发展规划》。加快新兴县省级全域旅游示范区建设，加快六祖故里旅游度假区创建国家5A级旅游景区，推动云城区创建省级全域旅游示范区。深入挖掘罗定长岗坡、南江古驿道等资源优势，培育一批特色旅游景区。办好中国农民丰收节和云浮乡村旅游节等活动，推动民宿经济发展。

加快推进乡村人才振兴。加快培育一批新型职业农民，加强乡村专业技术人才、技能人才、基层干部队伍建设。积极引导各类人才向乡村集聚，继续引进一批规划、旅游、农业等专业人才。鼓励外出乡贤助力家乡建设。

统筹推进脱贫攻坚。全面落实《打赢脱贫攻坚战三年行动实施方案》，完成年度脱贫任务。继续实施整镇推进产业扶贫，提高脱贫稳定性。强化扶贫资金管理，织密筑牢“三保障⑪”兜底保障网。

三、全面提升中心城区首位度，夯实高质量发展的重要载体

优化提升老城区。全力创建全国文明城市。年内建设65个提升市中心城区首位度项目。加快推进浩林西路、东方路、富民路等市政道路贯通，启动富乐路、翠石路北段等8段市政道路建设。加快推进“三旧”改造项目建设。完成英东体育场地下人防工程、蟠龙天湖景区升级改造工程。加快推进世纪大道风景带提升工程、南山河“一河两岸”风情带升级工程、南山森林公园三期建设等城市管理项目。加快建设一批城市综合体、文旅综合体。开展新一轮全市国土空间总体规划编制。推进海绵城市试点建设和地下综合管廊建设。

加快建设云浮新区。加快推进市档案馆、图书馆、美术馆、地方志馆和云安区中医院等建设。加快推进中央商务区建设，完成云浮东站站前广场建设。加快东部快线、西部快线、新城快线二期等道路建设。推动绿地集团、黄金珠宝智能制造产业园等项目落地。推动云城与新城协同创新发展，云安与新城融合发展。

优化道路交通网络。全市交通基础设施建设计划投资51.8亿元。汕湛高速清云段和高恩高速年内建成通车，完成广梧高速云浮东出入口交通组织及景观升级改造项目一期工程建设。加快推进怀阳高速二期、罗信高速等重点交通基础设施项目建设，着力抓好云浮新港等西江沿线码头扩能升级。加快开展广湛高铁、南深高铁前期筹备工作，推进开设广梧高速云浮西出入口前期工作。抓好国道324线改线等市域交通项目建设。加快省道、农村公路升级改造，促进与干线快速交通连接成网，实现城乡交通互联互通。

四、加强生态文明建设，厚植高质量发展底色

坚持保护原生态。科学划定永久基本农田、城镇开发边界和生态保护红线。启动涉环保容量及产业发展规划。加强森林防火灭火工作，全市森林火灾受害面积控制在0.5‰以下。完善环境联动执法和长效监管机制，全面落实网格化监管。

大力治理病生态。切实抓好环保督察整改。打好蓝天保卫战。全年空气质量全面达到国家二级标准，完成省下达的大气污染防治目标任务。打好碧水攻坚战。扎实推进“让广东河更美”大行动，继续全面抓好南江、新兴江、南山河“三水”整治，确保地表水省考断面优良水体比例达100%，西江西湾、都骑断面水质持续保持Ⅱ类，松云断面达到Ⅲ类。加快污水处理设施和配套管网建设，实现建制镇污水处理设施全覆盖。完成30宗中小河流治理、3宗病险水库除险加固工程建设任务。打好净土防御战，加强对石材废渣、矿渣、建筑垃圾等固体废物和危险弃废物的处置。

加快培育新生态。加快创建国家森林城市。落实“绿美南粤行动计划”，建设森林乡村85个，全年计划完成造林更新16.17万亩，新建生态景观林带10公里，完善提升生态景观林带19.5公里，森林覆盖率达到67.15%以上。

五、用好改革开放关键一招，激发高质量发展内生动力

深入推进营商环境综合改革。继续推动“放管服”改革十大行动计划落地见效，在优化企业开办、优化不动产登记、优化工程建设项目审批、方便企业获得水电气、便捷获得信贷、优化企业纳税服务、提升企业跨境贸易和投资便利化、审批服务便民化、县域营商环境监测评价、优化提升营商环境专项督查等10个方面加大改革探索力度。推进电力市场化交易和燃气终端销售价格改革，切实降低实体企业运营成本。全面推行商事登记全程电子化，进一步推广电子营业执照等涉企电子证照应用。深入推进“信用云浮”建设。加快推进“数字政府”建设,推动政务服务“一门、一窗、一网”办理，大力推广“粤省事·看云浮”等“指尖办事”便民服务。

深化重点领域和关键环节改革。全面完成市县机构改革。深化事业单位改革，加强事业单位登记管理。深化医药卫生体制改革，加快建立现代医院管理制度。继续推进国有林场改革，确保通过国家验收。继续深化“多规合一”，完善相关配套标准和机制。加快云浮农商行和郁南农商行改制组建进程。严格防控金融债务风险，确保不发生系统性、区域性金融风险。

积极扩大对外开放。积极融入“一带一路”建设，主动对接粤港澳大湾区发展，做活“区域融入”文章，着力把云浮打造成大湾区的“后花园”。深入推动投资和贸易便利化，支持跨境电商业务发展。落实省、市“外资十条”等政策措施，优化外商投资企业登记备案流程，全面实施商务备案和工商登记“一口办理”。全面推行国际贸易“单一窗口”，优化“无水港⑫”通关模式。支持石材、不锈钢制品、农产品等优势企业“抱团出海”，开拓多元化外贸市场，增强风险防范能力。继续办好云浮国际石材科技展览会、广东云浮·罗定稻米节、郁南无核黄皮节等品牌展会。

六、着力保障和改善民生，让高质量发展成果更多更好惠及群众

办好十件民生实事，努力让人民群众有更多获得感和幸福感。

优先发展教育事业。加快广东药科大学云浮校区二、三期建设。支持罗定职业技术学院扩容提质和广州华立科技职业学院云浮校区升本工作。谋划创建广东云浮（新兴）中医药职业学院。全面实施素质教育，加快补齐义务教育发展短板，落实中小学校建设三年行动方案。加快发展学前教育，加大规范化幼儿园和普惠性幼儿园建设力度。推进特殊教育发展。办好云浮开放大学。

加快健康云浮建设。加快市区域医疗中心建设，加大重点专科建设力度。推进基层医疗卫生服务能力建设，各县（市、区）启动新建一家医疗机构。继续抓好县级人民医院、中医院、妇幼保健院升级改造，确保罗定市、新兴县、郁南县第二人民医院建成投入使用。加强中医名院、名科、名医建设，加快创建国家中医药综合改革试验区。全面开展医联体建设，加快民营医疗机构发展。抓好疾病防控和卫生应急工作。落实计划生育目标管理责任制，做好优生优育服务。

提升就业创业和社会保障水平。年度新增就业1.8万人，帮扶800名就业困难人员实现再就业，城镇登记失业率控制在4%以内。推进市、县两级创业孵化基地建设，实施乡村创业引领行动。统筹推进医保制度改革，稳步提升社保待遇水平。切实保障农民工劳动报酬权益。继续推进农村特困人员供养服务机构社会化改革，完善养老服务体系建设。

大力发展文化体育事业。完善升级行政村综合性文化服务中心、“扫黄打非”基层站点规范化标准化建设。推动国恩寺等省级文物保护单位申报国家级文物保护单位。大力发展文化产业，加强与省文化和旅游厅、省广轻控股集团合作，共同打造中国石艺之都，加快建设南江文化旅游示范区。广泛开展全民健身活动。加快建设市体育公园，升级改造3个社区体育公园。举办云浮市首届击剑全国公开赛、首届马拉松赛、首届少儿轮滑展示大赛等体育赛事。

全面提升社会治理水平。纵深推进扫黑除恶专项斗争，加快建设“云浮智慧治理云图⑬”，争取申报建设全国社区治理和服务创新实验区、农村社区治理实验区。加强信访工作联席会议制度建设。加快基层司法所规范化建设和公共法律服务平台建设，打造市、县、镇、村四级法治宣传教育基地。全面推动安全生产责任落实，坚决遏制重大安全事故发生。严格落实食品安全“党政同责”，加快云浮市中药检测平台建设。深入开展打击制售假冒伪劣商品行动。推动军民深入融合发展，持续深化拓展全民国防教育，稳步推进国防动员和后备力量建设，持续征集输送优质兵员。

七、加强政府自身建设，以新担当新作为推动高质量发展

加强政治建设。坚持和加强党的全面领导，严格落实全面从严治党主体责任。以政府机构改革为契机，加快政府职能转变，牢记职责使命，切实增强履职能力，不断提高政务服务水平。

坚持依法行政。深入推进法治政府建设。依法接受市人大及其常委会监督，自觉接受市政协民主监督，主动接受社会和舆论监督，办理好人大代表建议和政协委员提案。完善行政决策机制，“三重一大”事项⑭坚持集体决策。完善政府立法机制，科学制定和开展重点领域政府立法。全面推进政务公开。全面落实“谁执法谁普法”责任制。

加强能力建设。各级政府领导干部要养成坚持学习、深入调研的好习惯，努力成为解决问题的能手。要具有发现问题的敏锐、正视问题的清醒、解决问题的自觉，把落实工作的切入点放到人民群众所需所盼所急上，放到改革发展的瓶颈、掣肘、难点上，不断开创工作新局面。完善政府效能考核，健全容错纠错和正向激励机制，对庸政懒政怠政行为严肃问责，为干部大胆创新、踏实干事撑腰鼓劲，大力营造干事创业的良好氛围。

坚持廉洁从政。深入推进政府系统党风廉政建设和反腐败斗争。严格执行中央八项规定及其实施细则精神，大力整治各种隐形变异“四风问题”，在整治形式主义、官僚主义上下更大功夫。加大对重大政策措施贯彻落实、公共资金、政府投资项目的审计力度，强化对领导干部经济责任审计、自然资源资产离任（任中）审计、生态环境审计、精准扶贫精准脱贫审计的跟踪和整改落实。严格控制“三公”经费和一般性支出，把有限的资源和财力用在推动发展、改善民生上。着力构建亲清新型政商关系，坚决惩治发生在群众身边的不正之风和腐败问题，铲除滋生黑恶势力的土壤，创造公平、公正的发展环境。

各位代表，今年发展的目标任务已经明确，关键在于抓落实。群之所为事无不成，众之所举业无不胜！让我们紧密团结在以习近平同志为核心的党中央周围，在省委、省政府和市委的坚强领导下，齐心协力，积极作为，真抓实干，为打造“两新一前列”、推进“美丽云浮、共同缔造”而努力奋斗！
